# Type VII secretion system and its effect on group B Streptococcus virulence in isolates obtained from newborns with early onset disease and colonized pregnant women

**DOI:** 10.3389/fcimb.2023.1168530

**Published:** 2023-07-21

**Authors:** Yulia Schindler, Galia Rahav, Israel Nissan, Gal Valenci, Miriam Ravins, Emanuel Hanski, Dana Ment, Dorit Tekes-Manova, Yasmin Maor

**Affiliations:** ^1^ Microbiology Laboratory, Mayanei Hayeshua Medical Center, Bney Brak, Israel; ^2^ The Sackler School of Medicine, Tel Aviv University, Tel Aviv, Israel; ^3^ Infectious Disease Unit, Sheba Medical Center, Ramat-Gan, Israel; ^4^ National Public Health Laboratory, Ministry of Health (Israel), Tel-Aviv, Israel; ^5^ Department of Microbiology and Molecular Genetics, The Institute for Medical Research, Israel-Canada (IMRIC), Faculty of Medicine, Jerusalem, Israel; ^6^ Department of Plant Pathology and Weed Research, Plant Protection Institute, Agricultural Research Organization, Volcani Institute, Rishon LeZion, Israel; ^7^ Infectious Disease Unit, Wolfson Medical Center, Holon, Israel

**Keywords:** *Streptococcus agalactiae*, type 7 secretion system, pregnancy, sepsis, neonate, early onset disease, virulence

## Abstract

**Introduction:**

GBS may cause a devastating disease in newborns. In early onset disease of the newborn the bacteria are acquired from the colonized mother during delivery. We characterized type VII secretion system (T7SS), exporting small proteins of the WXG100 superfamily, in group B Streptococci (GBS) isolates from pregnant colonized women and newborns with early onset disease (EOD) to better understand T7SS contribution to virulence in these different clinical scenarios.

**Methods:**

GBS genomes [N=33, 17 EOD isolates (serotype III/ST17) and 16 colonizing isolates (12 serotype VI/ST1, one serotype VI/ST19, one serotype VI/ST6, and two serotype 3/ST19)] were analyzed for presence of T7SS genes and genes encoding WXG100 proteins. We also perform bioinformatic analysis. *Galleria mellonella* larvae were used to compare virulence between colonizing, EOD, and mutant EOD isolates. The EOD isolate number 118659 (III/ST17) was used for knocking out the essC gene encoding a membrane-bound ATPase, considered the driver of T7SS.

**Results:**

Most GBS T7SS loci encoded core component genes: essC, membrane-embedded proteins (essA; essB), modulators of T7SS activity (esaA; esaB; esaC) and effectors: [esxA (SAG1039); esxB (SAG1030)].Bioinformatic analysis indicated that based on sequence type (ST) the clinicalGBS isolates encode at least three distinct subtypes of T7SS machinery. In all ST1isolates we identified two copies of esxA gene (encoding putative WXG100proteins), when only 23.5% of the ST17 isolates harbored the esxA gene. Five ST17isolates encoded two copies of the essC gene. Orphaned WXG100 molecule(SAG0230), distinct from T7SS locus, were found in all tested strains, except inST17 strains where the locus was found in only 23.5% of the isolates. In ST6 andST19 isolates most of the structure T7SS genes were missing. EOD isolates demonstrated enhanced virulence in *G. mellonella* modelcompared to colonizing isolates. The 118659DessC strain was attenuated in itskilling ability, and the larvae were more effective in eradicating 118659DessC.

**Conclusions:**

We demonstrated that T7SS plays a role during infection. Knocking out the essC gene, considered the driver of T7SS, decreased the virulence of ST17 responsible for EOD, causing them to be less virulent comparable to the virulence observed in colonizing isolates.

## Introduction

Group B streptococcus (GBS) also known as *Streptococcus agalactiae* is a commensal bacterium that belongs to the human microbiota colonizing the gastrointestinal and genitourinary tract (Shabayek and Spellerberg, 2018). In most cases the colonization in humans is harmless, but GBS can also cause severe disease (Dermer et al., 2004; Filkins et al., 2021). An important manifestation of GBS disease is neonatal sepsis and meningitis (Dermer et al., 2004). Early-onset disease (EOD) in the newborn is a devastating disease that results from vertical transmission of GBS from colonized mothers through contaminated amniotic or vaginal secretions to her newborn. GBS isolates can be divided into10 distinct serotypes (Ia; Ib; II-IX) based on a serological reaction directed against the polysaccharide capsule (Shabayek and Spellerberg, 2018). Most human GBS isolates can be clustered into six major sequence types (STs) (Shabayek and Spellerberg, 2018).

GBS has a variety of putative virulence factors that facilitate its ability to cause disease, some of which have been identified and characterized (Nizet and Rubens, 2014).

Bacterial pathogens utilize a multitude of methods to invade mammalian hosts, damage tissue sites, and escape the immune system (Vornhagen et al., 2017). One essential component for many bacterial pathogens is secretion of proteins across phospholipid membranes (Green and Mecsas, 2016). Type VII secretion system (T7SS) is a specialized secretion system in Gram positive bacteria first discovered in *Mycobacterium spp*,. It is responsible for the export of small proteins that are members of the WXG100 superfamily (Rivera-Calzada et al., 2021). In *Mycobacterium tuberculosis* T7SS plays an important role in bacterial virulence and persistence of infection (Gray et al., 2016; Aly et al., 2017; Famelis et al., 2019). Analogous substrates and some components of these systems have also been identified in several other Gram-positive organisms, including *Staphylococcus aureus*, *Streptococcus pyogenes, Streptococcus pneumoniae* and *Bacillus anthracis* (Way and Wilson, 2005; Garufi et al., 2008; Cao et al., 2016).

There are commonalities and differences between the T7SS of *Actinobacteria* and *Firmicutes* (Costa et al., 2015; Rosenberg et al., 2015). A membrane-embedded ATPase of the FtsK/SpoIIIE family termed EssC is found in all T7SSs. In both systems the protein shares a similar overall topology, with two transmembrane domains that are usually followed by three P-loop ATPase domains at the C-terminus, that energize substrate secretion. The second feature of T7SS is the presence of canonical substrate WXG100 protein EsxA [named for its 100 amino acid sequence and central Trp-X-Gly (WXG) motif], which is secreted by T7SS (Tran et al., 2021). The ATPase domain of EssC interacts with WXG100 protein substrates, through a signal sequence (Pallen, 2002).

In *Mycobacteria*, EsxA homologues are secreted as heterodimers with EsxB (LXG-domain containing protein) (Renshaw et al., 2005; Poulsen et al., 2014), whereas in Firmicutes EsxA is secreted as a homodimer (Simeone et al., 2015; Cao et al., 2016). The T7SS is encoded by the *ess* locus. In addition to EsxA and EssC, further integral and peripheral membrane proteins are encoded by the locus EsaA, EssA, EssB and EsaB. In *S. aureus* these membrane proteins are essential components of the secretion machinery (Cao et al., 2016; Lai et al., 2017). Additionally, increasing number of reports have shown a role for the T7SS and/or EsxA in the pathogenesis of several other Gram-positive bacteria (Cao et al., 2016; Taylor et al., 2021; Tran et al., 2021) However there is insufficient data regarding the structure and distribution of T7SS in clinical GBS strains. Recently the structure of T7SS in GBS strains was characterized and four T7SS subtypes based on the C-terminus of the ATPase EssC were identified (Spencer et al., 2021). The genetic diversity of the T7SS in GBS isolates was also identified (Zhou et al., 2022) but the clinical significance of this secretion system in GBS is still unknown.

We recently demonstrated that in a population of orthodox Jews treated at Maayaney Hayeshua Medical Center (MHMC) serotype III [sequence type (ST)17] was the most common serotype in EOD cases while serotype VI (ST1) was the prevalent serotype among colonizing isolates (Schindler et al., 2020; Schindler et al., 2023). This prompted us to search for the presence and structure of the T7SS locus among these clinical GBS isolates and to assess the effect of the T7SS on the virulence of isolates causing colonization and isolates causing invasive disease (EOD).

## Materials and methods

### Bacterial strains and growth conditions

We studied all available EOD isolates obtained from blood cultures of neonates (n=17) and a random sample of colonizing isolates (n=16) obtained during routine screening from the vagina of asymptomatic pregnant women during hospitalization at MHMC. The distribution of the colonizing isolates was similar to that seen in a larger epidemiological study we previously performed (Schindler et al., 2023). The description of the isolates used in this work is presented in the supplement (**Table S1A**). The study was approved by the hospital’s Internal Review Board (approval number 0023-18-MHMC) and we received a waiver of informed consent as no clinical data was reported in this study.

GBS isolates were preserved in sterile Brain Heart Infusion (BHI) broth with 15% glycerol (HyLabs, Israel) at −70°C for long-term storage and at 4°C for short-term maintenance. For quantitative qRT-PCR analysis, GBS strains were grown in BHI medium (Hylabs, Israel) at 37°C with 5% CO2 under shaking conditions*. Escherichia coli* was grown aerobically in Luria–Bertani (LB) (Hylabs, Israel) at 37°C. Antibiotics were added: for GBS 250 µg/ml kanamycin (Km), and 1 µg/ml erythromycin (Em); for *E. coli*: 100 µg/ml ampicillin (Amp), 500 µg/ml Em and 50 µg/ml Km (St Louis, MO, USA).

### Bioinformatic analysis of T7SS genes in GBS clinical isolates

Genomic libraries of clinical GBS isolates were prepared using Nextera XT kits (Illumina, San Diego, CA) and sequenced using the Illumina MiSeq Reagent Kit v3 (600-cycle). The reads obtained for each sample were trimmed and the quality of the Fastq reads was examined using the Fastq Utilities Service, and finally assembled by SPAdes using the PATRIC website (Wattam et al., 2017). The presence of T7SS genes was identified using web-resources: the bacterial bioinformatics database and analysis resource of PATRIC website (https://www.patricbrc.org/) and NCBI BLASTp (available at www.ncbi.nlm.nih.gov/blast/). A high-quality representative genome of *S. agalactiae COH1* (ST17) was used as reference for EOD isolates (Da Cunha et al., 2014) and 2603V/R ATCC BAA611 (ST19) was used as reference for colonizing isolates (Tettelin et al., 2002). We characterized the structure and membrane topology of genes using the HHpred interactive server. We identified genes that encode WXG100 proteins presumably secreted by T7SS, by detecting the presence of signal peptides using Phobius and SignalP tools. We compared the structure and the presence of T7SS effectors among ST17 and ST1 GBS isolates. To assess changes in conservation between the various isolates we compared them to reference strains and assessed the degree of protein homology. The protein sequence-based genome comparisons were done using bidirectional BLASTP by the PATRIC server. This tool provides information about conserved genomic contexts, and the presence of insertions or deletions. We compared the genomic coding sequences (CDSs) of ST17 strains and ST1 strains to the reference strains. The results are displayed with color-coding for protein percent identity relative to the best hit on the reference genome.

### Generation of a knockout GBS strain

The EOD 118659 (serotype III/ST17) strain (**Table S1A**) was used for knocking out the *essC* gene encoding a membrane-bound ATPase, considered the driver of T7SS. We chose this isolate as it contains only one copy of the *essC* gene, missing *esxA* gene and was previously found sensitive to all tested antibiotics (penicillin, erythromycin and clindamycin) (**Table 1**).

**Table 1:** Protein percent homology of the T7SS components among different GBS isolates.

Gene name		*esxA*	*esxB*	*esaC*	*essC*	*essC*	*essB*	*esaB*	*essA*	*esaA*	*esxA*
ST type	Sample #	SAG0230	SAG1031	SAG1032	SAG1033	SAG1034	SAG1035	SAG1036	SAG1037	SAG1038	SAG1039
**ST1**	M38307	99.9	34.6	61	90		98.1	99.9	96.4	98.1	99.7
	M38421	99.9	34.6	61	90		98.1	99.9	96.4	98.1	99.7
	M38742	99.9	34.6	61	90		98.1	99.9	96.4	98.1	99.7
	M38914	99.9	34.6	61	90		98.1	99.9	96.4	98.1	99.7
	M39081	87.8	34.6	61	90		98.1	99.9	96.4	96.4	99.7
	M39881	99.9	34.6	61	90		98.1	99.9	96.4	98.1	99.7
	M40042	99.9	34.6	61	90		98.1	99.9	96.4	98.1	99.7
	M40064	99.9	34.6	61	90		98.1	99.9	96.4	98.1	99.7
	M40942	99.9	34.6	61	90		98.1	99.9	96.4	98.1	99.7
	M41387	99.9	34.6	61	90		98.1	99.9	96.4	98.1	99.7
	M41827	99.9	34.6	61	90		98.1	99.9	96.4	98.1	96.4
	W19655	99.9	34.6	61	90		98.1	99.9	96.4	98.1	96.4
**ST19**	M38346	99.9									87.7
	M38603	99.9									87.7
	M40083	99.9									87.7
**ST6**	M38839										
**ST17**	101298	99.9	40		99.5		99.5	99.9	99.9	99.8	99.7
	106704		40		99.9		99.9	99.9	99.9	99.5	
	111236		40		99.8		99.8	99.9	99.9	99.8	
	112109		40		99.9		99.9	99.9	99.9	99.8	
	112767		40		99.9	95	99.9	99.9	99.9	99.8	
	117690		40		99.8		99.8	99.9	99.9	99.8	
	118022		40		99.8	95	99.8	99.9	99.9	99.8	
	118659		40		99.8		99.8	99.9	99.9	99.8	
	121684	99.9	40		99.9		99.9	99.9	99	99.8	
	123494		40		99.9		99.8	99.9	99.9	99.8	
	127743	99.9	40		99.8		99.8	99.9	99.9	99.8	87.7
	127946				99.9	95	99.8	99.9	99.9	99.8	
	129618		40		80		99.8	99.9	99.9	99.8	
	134924	99.9	40		99.9	95	98.8	99.9	99.9	99.8	87.7
	135217		40		99.9	96.4	98.8	99.9	99.9	99.5	
	139904		40		99.9		98.8	99.9	99.9	99.8	
	139934	99.9	40		99.8		99.8	99.9	99.9	99.8	99.7

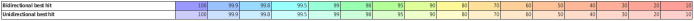

To assess changes in conservation between the various isolates we compared them to reference strains and assessed protein homology between them. This was done by using heatmaps to indicate protein sequence homology of T7SS-associated genes across the various ST types. Color intensity based on protein percent identified ST17 strains relative to a reference GBS genome *S. agalactiae* COH1 (ST17) and the colonizing isolates STs relative to *S. agalactiae* 2603 V/R (ST19). Changes in conservation relative to the reference genome were indicated by color going from blue, representing the highest protein sequence similarity, to red, representing the lowest protein sequence similarity.

A deletion mutant was created using the temperature sensitive plasmid pJRS233 with a kanamycin resistance gene, *Km* in the knockout construct, as previously described (Jiang et al., 2008) (**Table S1B**). Briefly, the flanking regions of the *essC* gene of GBS118659 were amplified using EssC-KO-F and EssC-KO-R primer pairs (**Table S2**). The 3809-bp PCR product was purified and cloned into pGEM-T-Easy (Promega, Medison WI, USA) to yield pGEM: *essC*. The plasmid was transformed to *E. coli* DH5α by electroporation, plated on LB plates containing ampicillin 100 μg/mL with x-gal and IPTG, and allowed to grow for one day at 37°C. Positive transformants (white colonies) were confirmed by PCR and sequencing. Restriction of pGEM: *essC* plasmid with HpaI and KpnI, releases a 2934 fragment of *essC* leaving 411bp and 465bp of *essC* on each side- for homologous recombination to the GBS chromosome. Next, the digested plasmid was treated with Klenow enzyme and ligated with a 2043bp fragment of *SmaI* digested Ωkm cassette (kanamycin resistance cassette flanked by Ω elements). The pGΔEssCΩKm plasmid was transformed to *E. coli* DH5α by electroporation, plated on LB plates containing kanamycin (Km) - 50 μg/mL. Positive transformants were confirmed by PCR and sequencing. The plasmid was restricted with NotI (releasing a 2954 bp fragment of ΔEssCΩKm flanked by *essC* sequences) and ligated into NotI digested pJRS233 plasmid (a temperature-sensitive shuttle vector). The pJΔ: EssC: ΩKm plasmid was transformed to *E. coli* DH5α by electroporation, plated on LB plates containing Km50/Em500. Positive transformants were confirmed by PCR. 3-7µg of pJΔ: EssC: ΩKm plasmid was transformed into competent GBS cells (strain 118659) by electroporation (25 µF, 400ohms, 1.75 KV) and bacteria were plated on THY plates containing erythromycin 1 μg/mL. Erythromycin-resistant transformants were then cultured under non-permissive temperature to select for single cross-over recombinants, followed by serial passage in antibiotic-free BHI and screening for double cross-over deletion mutants by PCR. The schematic presentation of pJRS EssC omega Km plasmid which was transformed to *E. coli* DH5α is shown in **Figure S1**.

Deletion of the gene was confirmed by PCR amplification of the regions spanning the deleted fragment using the EssC-KO-F and EssC-KO-R primers, primers for kanamycin resistance, and a pair of primers from inner part of *essC* gene (Conf-KO-essC), which should have been replaced by the omega kanamycin cassette (**Table S2**). The absence of any secondary site mutations was confirmed by whole genome sequencing.

### *In vitro* phenotypes of 118659 *ΔessC* mutant and 118659 Wild type strains

The 118659 Wild type (WT) and 118659Δ*essC* (mutant) strains were grown overnight in BHI medium, 1:20 diluted in fresh BHI medium at the zero-time point and incubated at 37°C + 5% CO2 under shaking conditions. The optical density (OD) at wavelength 600 nm of each group, was measured for 8 hours (achieving the stationary phase). Each experiment was repeated three times. The WT and mutant strains were cultured on blood agar (Hylabs, Israel) and incubated at 37°C + 5% CO2 for 24 hours to observe hemolytic activity.

### *Galleria mellonella in vivo* model

*Galleria mellonella* larvae were obtained from the Volcani center (Dr. Dana Ment laboratory, Entomology department), kept in darkness at room temperature, and discarded after one week following arrival. Healthy larvae measuring from 2-2.5 cm were used for all experiments. Injections were done using INSUMED 29G insulin syringes (Pic solution) (Six et al., 2019). For each experiment groups of 10 larvae were injected with 10 µl of serial dilutions of bacterial suspension. A control group including five larvae were inoculated with PBS for control of motility change caused by physical injury or infection by a contaminant. Experiments were repeated twice. After injection, larvae were observed at room temperature for 15–30 min to ensure recovery and were stored in Petri dishes in the dark at 37°C. Survival of infected larvae was monitored for 72 hours post infection (p.i). The larvae were considered dead when non-responsive to touch.

### Survival assay

GBS isolates were grown to an OD 0.4-0.6 in BHI (∼1 × 10^9^ colony forming units [cfu] per ml), washed and resuspended in PBS (Hylabs, Israel), and then diluted prior to injection. Cells were washed twice in sterile PBS and diluted to the desired inoculum. The starting inoculum was confirmed through serial dilution, plating on blood agar plates (Hylabs, Israel) just before administration for CFU counting. To determine the dose required to kill 50% of the population (LD_50_), four groups of 10 larvae were injected with 20 µl of serial dilutions of bacterial suspension as described above. Kaplan–Meier curves and the log-rank test were used to assess survival (SPSS). LD_50_ was calculated using Probit and differences in LD_50_ between different isolates were assessed by the Mann-Whitney test.

### *In vivo* GBS growth curve

Groups of 10 larvae were infected with 118659 (WT) and 118659*ΔessC* (mutant) strains and monitored for 72 hours. At fixed time points (8, 24, 48, and 72 h p.i.), larvae were kept at −20°C for 10 min before being transferred to Eppendorf containing 100 µL of sterile PBS, homogenized by mechanical disruption, serially diluted. CFU counts from homogenized infected larvae were determined by the viable plate count method using selective Chromo Strep B plates (Hylabs, Israel).

### Competition assay

To distinguish between WT and mutant GBS strain, we induced resistance to streptomycin (Sm) in the WT strain by culturing and passing it several times under high streptomycin concentrations. GBS strains were grown to log phase (OD_600 = _0.4) for 3-4 hours, washed and resuspended in PBS. Mutant strains were mixed with the parental (WT) at a 1:1 ratio. Ten microliters of the mixed culture (~1 × 10^7^ total CFU) were injected into each larva and larvae were then incubated for 24 h at 37°C. We chose 24 hours as this was long enough for the infection to become established but short enough to preclude total larval mortality. The larvae were then rinsed in 70% ethanol followed by sterile water to help minimize contamination by surface bacteria before being homogenized in PBS by mechanical disruption. Homogenates were plated on BHI and BHI-antibiotic plates (Hylabs, Israel) (streptomycin (SM500) for WT and kanamycin (Kan250) for mutant strain) and the CFU recovered for each strain was calculated.

### Monitoring *G. mellonella* larvae

*G*. *mellonella* larvae were monitored daily for activity, silk production (cocoon formation) and melanization (**Table S3**). Loh et al. (Tsai et al., 2016) developed these criteria to evaluate the health status of the larva during an infectious process and to assess subtle differences in virulence of different bacterial pathogens (Loh et al., 2013; Tsai et al., 2016; Ghigo et al., 2019; Vertyporokh and Wojda, 2020). An uninfected group of larvae and a group inoculated with saline were used as negative controls. A score was assigned to each observation, and an overall health index score was calculated for each larva.

### Clearance of mutant and WT strains by *G. mellonella*

*G. mellonella* larvae were injected with a sublethal inoculum (the closest dose to killing 15% of the larvae) ≈1 × 10^5^ CFU of 118659 (WT) strain and ≈1 × 10^6^ CFU 118659Δ*essC* (mutant) strain, monitored every hour for 7 hours and after 12 hours. At each fixed time point, three surviving larvae were randomly selected, kept for 15 min on ice and bathed in 70% ethanol and sterile water. The selected larvae were homogenized in 2 ml. For bacterial count serial dilution were performed and the homogenate was plated in blood agar (Hy-Labs, Israel) and selective Chromo Strep B plates (Hy-Labs, Israel).

### Transcriptional analyses

Quantitative RT-PCR analysis of *esaA*, *esxA*, *essA*, *essB*, and *essC* genes expression was performed as described previously (Spencer et al., 2021). Primers were designed using Primer3 Plus and Clone manager 9 professional edition, version 9.4 software. Primers were used at a final concentration of 0.4 μmol/L (**Table S4**). RNA was extracted from GBS cultures grown at 37°C to an exponential growth phase in BHI medium. RNA was purified using the Rneasy Mini kit (Qiagen) according to manufacturer instructions. Purified RNA was treated with the DNAse kit (HY-labs, Israel) according to manufacturer instructions. The RNA quality and concentration was assessed by NanodropTM and visually on a 2% E-Gel with SYBR safe (Invitrogen, Thermo) and visualized by E-Gel Power Snap Electrophoresis device (Invitrogen, Thermo Fisher).

cDNA was synthesized using the Hy-RT-PCR kit (HY-labs, Israel), according to manufacturer instructions. cDNA was diluted 1:150 to further reduce bacterial DNA contamination and qPCR was performed using Hy-SYBR power mix (HY-labs, Israel) and CFX96 Real-Time System (Biorad). RNA from three independent biological triplicates were analyzed and final cycle threshold for each strain was calculated (mean value of three experiments). Relative quantification of gene expression was performed using comparative 2^- ΔΔCT^. Results were normalized using *rpoB* gene as the housekeeping gene.

### Statistical analyses

Statistical analysis was performed using SPSS version 27.0 (SPSS Inc., Chicago, IL, USA). Statistical details of experiments, such as statistical tests used, experimental *n*, can be found in each figure legend. Significance was defined as p < 0.05.

## Results

Thirty-three GBS isolates were sequenced and studied, 17 from neonates with EOD and 16 from asymptomatic pregnant women. ST types were: ST17 (n=17) from neonates with EOD, ST1 (n=12), ST19 (n=3) and ST6 (n=1) from asymptomatic pregnant women.

### Identification of three GBS T7SS subtypes among various ST’s

**Figure 1:**
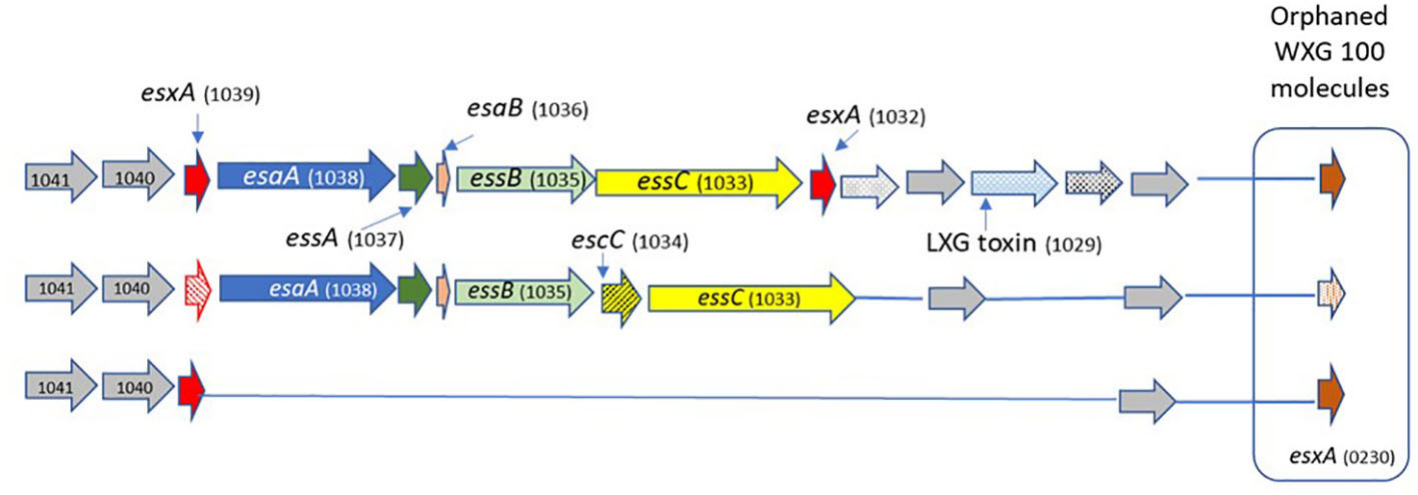
The schematic structure of the T7SS system in different GBS ST types. GBS T7SS encodes conserved machinery genes (*esaA*,*essA*,*esaB*,*essB*), but subtypes vary in the number of copies of *esxA* (red) and in putative downstream effectors, including WXG100-like proteins (brown), putative LXG toxins (light blue), and hypothetical proteins (gray). Arrows with patterns indicate genes which were not detected in all isolates.

We analyzed the structure of the T7SS locus compared to the reference genome *S. agalactiae COH1* (ST17) and the reference genome *S. agalactiae* 2603 V/R (ST19). We also analyzed the genomes of the clinical GBS isolates, using a Protein BLAST utility, to determine presence/absence of T7SS-associated proteins across various GBS sequence types (**Table 1**). We observed structures related to T7SS in all isolates except in colonizing ST19 and ST6 isolates (Warne et al., 2016) The difference between the isolates was in the presence of one or two *essC* genes (encoding FtsK/SpoIIIE-type ATPase), presence of the WXG100 protein-encoding genes and the presence of a putative LXG toxin/anti-toxin-encoding gene (*esxB*). Based on these results we suggested three subtypes of T7SS in GBS from different ST’s (**Figure 1**).

#### Subtype I (ST1, n=12)

ST1 isolates encode one copy of *essC* gene (SAG1033); two copies of the WXG100 protein-encoding gene, *esxA* (SAG1039) located upstream, SAG1032 is located downstream of the T7SS core genes, and another gene not directly linked to the T7SS locus (orphaned) (SAG0230). ST1 isolates encode the *esxB* gene (putative T7SS effector including an LXG-domain containing protein) downstream of the T7SS core genes.

#### Subtype II (ST17, n=17)

ST17 isolates have a smaller T7SS locus, and most of them lack genes encoding the WXG100 proteins (*esxA*). SAG1039 and SAG0230 were present in only 23.5% of isolates, and 23.5% of the isolates have another copy of the *essC* gene (SAG1034).

#### Subtype III (ST19, n=3; ST6 n=1)

All structural and regulatory T7SS genes are missing. *esxA* (SAG1039) and *esxA* (SAG0230) were present in all tested isolates.

### Expression levels of *essC* and *esxA* genes among EOD and colonizing GBS isolates

**Figure 2:**
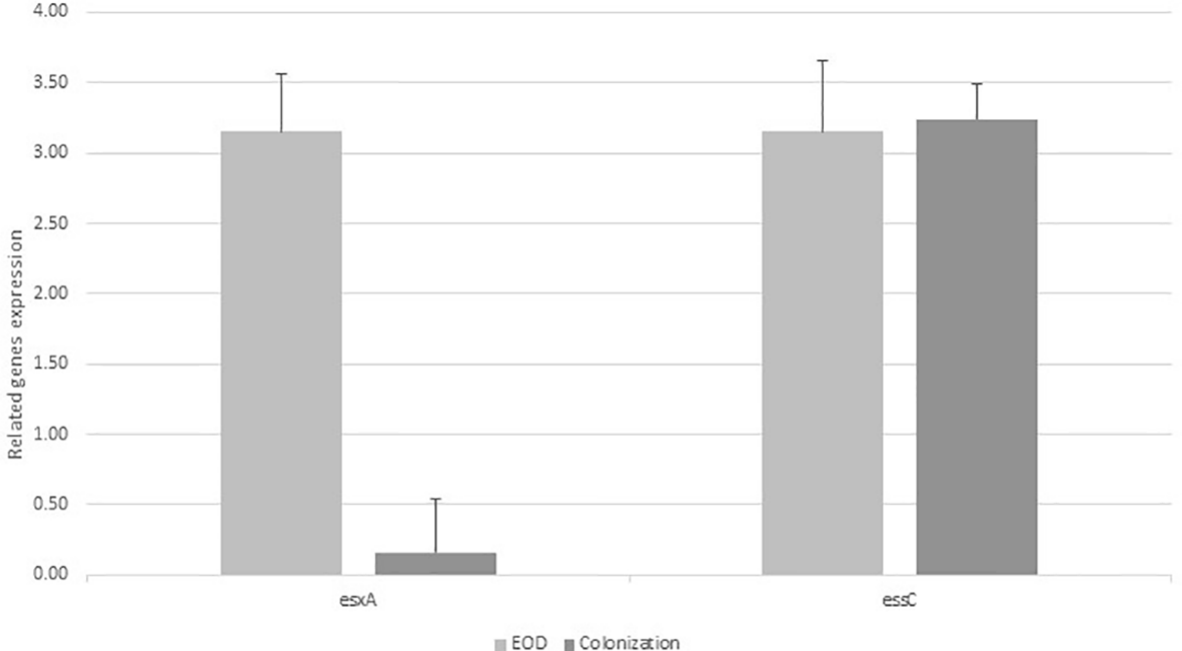
The expression of *esxA* and *essC* genes in early-onset disease (EOD) and colonizing isolates. Quantitative RT-PCR analysis was used to determine *esxA* and *essC* expression. Amounts of *esxA* and *essC* transcript expressed were assessed relative to *rpoB* gene expression in the reference strains. Data according to the expression of *essC* gene was collected from 8 experiments with EOD (101298,118659,121684, 123494, 127743,134924,135217, 139934) and 8 experiments with colonizing strains (M38307, M38421, M38742, M38914, M39081, M39881, M40042, M40064), and the expression of *esxA* was collected from four experiments with EOD strains (101298, 127743, 134924, 139934) and four experiments with colonizing strains (M38307, M38421, M38742, M38914), with 10 larvae per group for each experiment. In the figure we show values of one representative (mean of three independent biological triplicates) EOD strain and one representative colonizing strain).Difference in expression ratio were compared using unpaired t tests (p<0.001).

We analyzed the transcription of genes encoding the integral membrane bound ATPase protein *essC* (SAG1033) among EOD/ST17 (n=8) and colonizing/ST1 (n=8) GBS isolates. The expression of *esxA* (SAG1039) was tested among EOD/ST17 (n=4) and colonizing/ST1 (n=4) GBS isolates. The *essC* gene was expressed in all tested GBS isolates. However, *esxA* was weakly expressed among colonizing isolates compared to a significant expression in EOD isolates (**Figure 2**).

### GBS virulence (EOD and colonizing isolates) in *G. mellonella* model

*G. mellonella* larvae were used as an *in vivo* model of infection for GBS. We tested four ST17 EOD strains and four ST1 colonizing strains (**Table S1A**). All GBS isolates induced a dose-dependent response in the larvae that was reproducible for each isolate in three independent experiments (**Figures S2A, B**).

**Figure 3:**
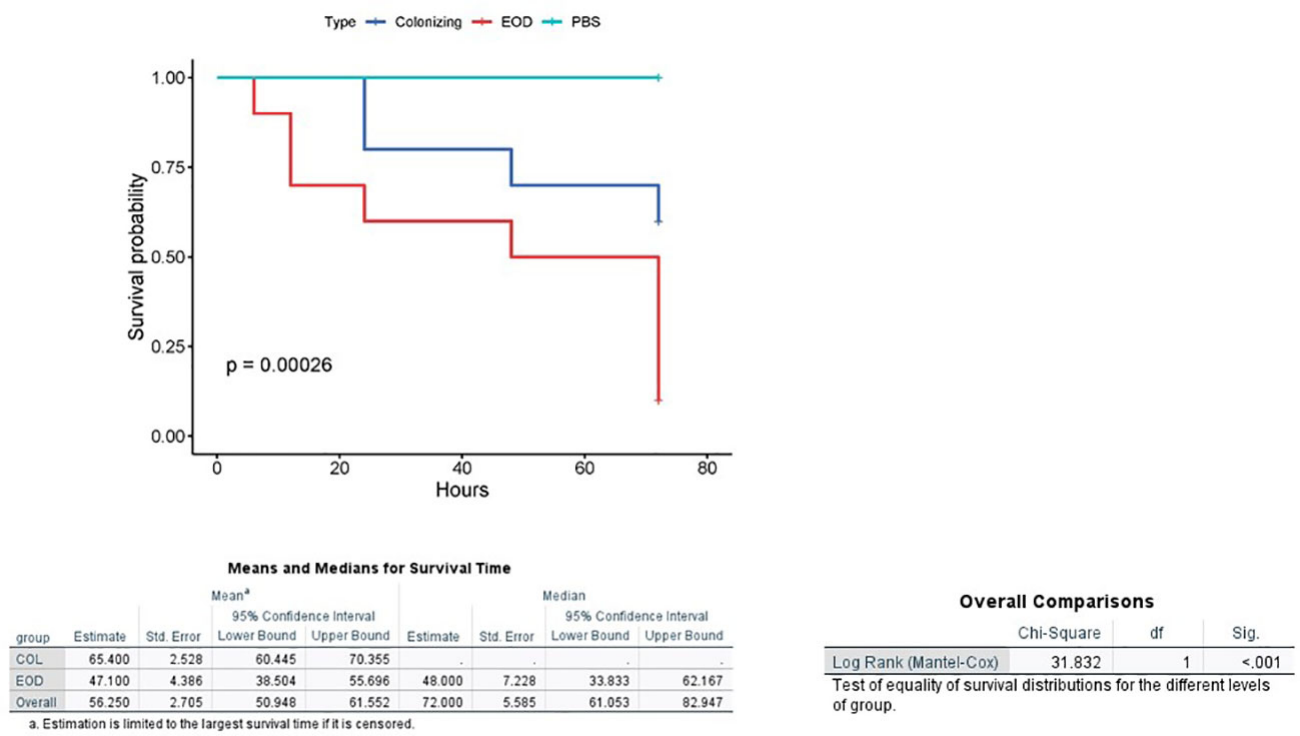
Kaplan-Meier survival plot of larvae challenged with EOD and colonizing (COL) isolates. A Kaplan–Meier survival plot of survival after infection with four ST17 EOD isolates (118659,121684, 123494, 127946) and four ST1 colonizing isolates (M38307, M38421, M38742, M38914). Each infection was repeated three times with 10 larvae for each experiment. PBS-injected larvae were used as a negative control, and all survived until the endpoint of the experiment.

We then injected varying doses of GBS isolates (EOD n=4, colonizing n=4) into each larva to compare the virulence of EOD and colonizing isolates after 72 hours by measuring the infecting dose killing 50% of the larvae (LD_50_) (**Figure S3**). LD_50_ values obtained for infection with EOD isolates (2.7x10^6^) were significantly lower (p<0.05) than those of colonizing (ST1) isolates (4.1x10^11^, this number was extrapolated as we could not kill 50% of the larvae in the colonizing isolates), indicating that an isolate associated with EOD has increased virulence in *G. mellonella* compared to colonizing strains. Twenty-four hours after infection with EOD isolates, only 60% of infected larvae survived, compared to 90% survival rate after infection with colonizing strains (**Figure 3**).

### Attenuation of 118659 EOD/ST17 isolate by *essC* knockout

To understand the role of *EssC* in virulence of GBS strains, we generated an isogenic *essC* mutant in the clinical isolate 118659 EOD/ST17. PCR analysis of the mutant 118659Δ*essC* produced bands with a different size than those observed for the 118659 WT strain (2900 bp versus 3300 bp), indicating that the *essC* gene was disrupted by the insertional mutagenesis of the kanamycin cassette (**Figure S4**). The insertion of kanamycin resistance gene was validated using primer pairs v-omega-Km1 and v-omega-Km2, in composition with EssC-KO primers located in both ends of original amplicon. The PCR analysis using these primers generated bands only in the mutant strain and were absent in the WT strain (**Figure S4**). According to variant analysis of the 118659 WT and 118659Δ*essC* (mutant) genomes against the reference genome, the difference between them relied only on the deletion of the *essC gene* and no additional mutations were identified.

In standard rich medium the Δ*essC* had a similar growth rate as the WT 118659 (**Figure S5**). Additionally, Δ*essC* did not show a growth defect or difference on hemolysis production when cultured in parallel with the WT strain. All tested strains had the same prototypical phenotype and displayed a narrow zone of beta-hemolysis on blood agar plate (**Figure S6**).

### Expression of core components of T7SS in 118659Δ*essC* (mutant) strain

**Figure 4:**
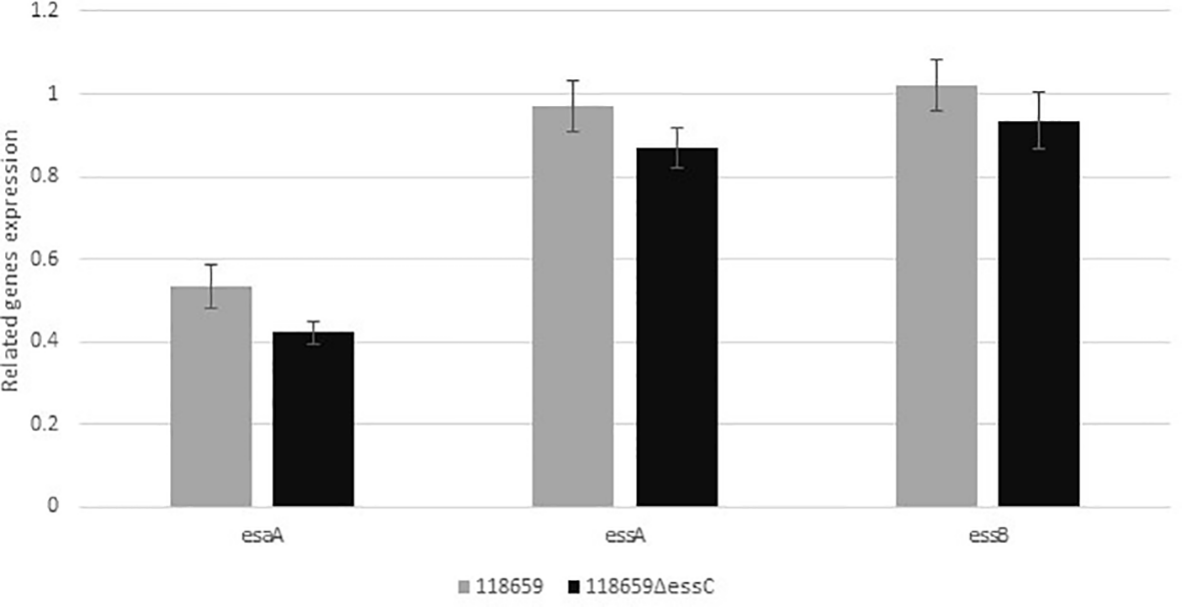
Relative gene expression of *esaA*, *essA*, *essB*, and *esaB* genes. Quantitative real-time RT-PCR analysis was used to determine the expression of genes belonging to the T7SS operon, located upstream to *essC* gene. The amount of each transcript were normalized to *rpoB* gene and expressed relative to that of the gene in the reference strain. Values represent the means of three independent RT-qPCR experiments (triplicates for each experiment were used). Difference in expression ratio were compared using unpaired t tests (p<0.0001).

We compared the gene expression of *esaA*, *essA*, and *essB*, located upstream to *essC* gene to study the influence of *essC* knock out on their activity. qRT-PCR analysis revealed similar levels of expression of tested genes among mutant and WT strains demonstrating that the activity of the whole T7SS locus was not disturbed by knocking out the *essC* gene (**Figure 4**).

### Contribution of *essC* gene mutation to GBS virulence in *G. mellonella in vivo* model

**Figure 5:**
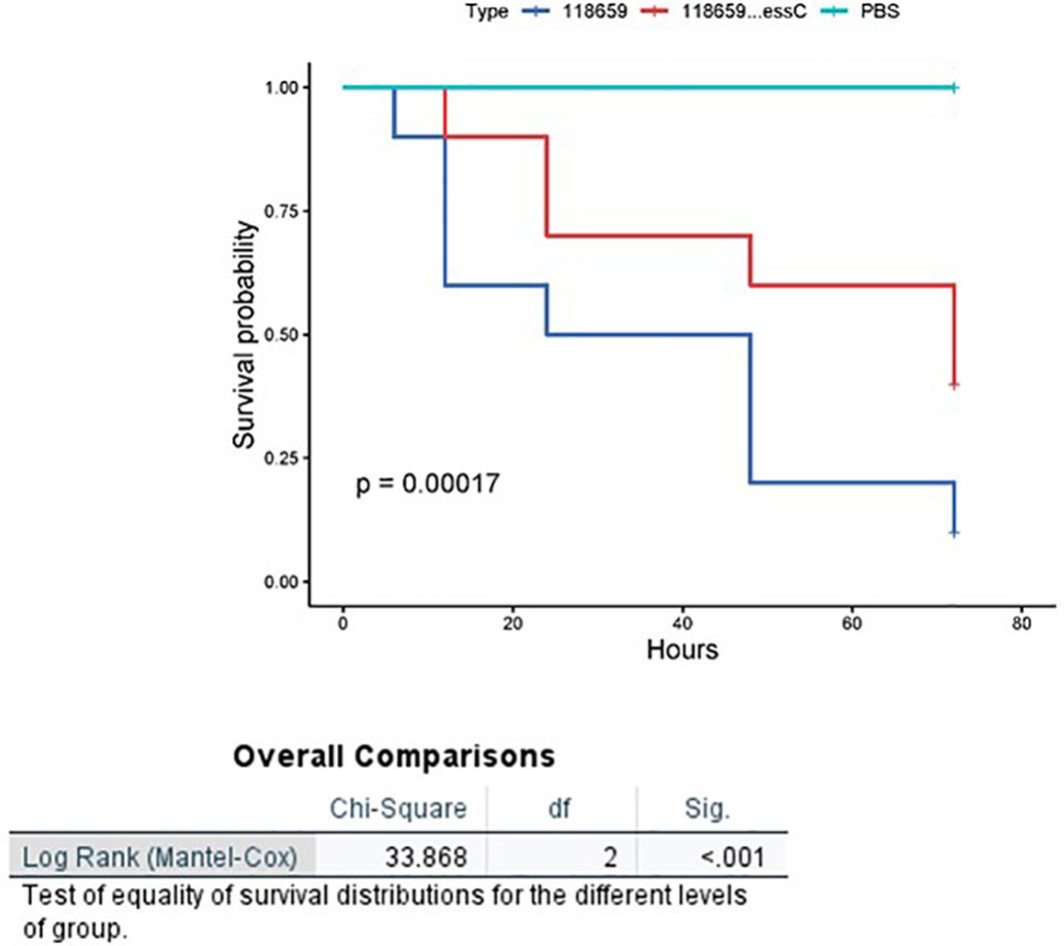
Kaplan-Meier survival curves of larvae challenged with EOD 118659 (WT) and mutant (MUT) strain (118659ΔEssC). Kaplan-Meier survival curves of larvae challenged with an inoculum of 10^7^ CFU of 118659 (WT), 10^8^ CFU of mutant strain (118659ΔEssC), and PBS (control). Each infection was repeated three times with 10 larvae for each experiment, (p < 0.05; log-rank test).

To assess the ability of the *G. mellonella* model to discern changes in virulence between the 118659Δ*essC* (mutant) and 118659 (WT) strain the infecting dose (LD_50_) for each strain was determined (**Figure S7c**). LD_50_ values obtained for infection with mutant strain were significantly higher than those of WT strain (4.1x10^9^compared to 2.7x10^7^, p < 0.01) indicating that in the *G. melonella* model the mutant strain is less virulent. Larval mortality appeared 6-8 hours after infection with both WT and the mutant isolates but increased progressively mainly in the WT strain. Larval mortality in the mutant inoculated group was significantly reduced (p = 0.03) compared to the WT strain (**Figure 5**). The larval survival rate after 72 hours post infection with the WT strain was 10%, compared to 40% with the mutant strain. In summary, the mutant strain had decreased ability to kill *G. mellonella*, indicating that the *essC* gene may play an essential role in GBS virulence.

### Kinetics of *in vivo* growth of WT and GBSΔ*essC* (mutant) strains

To monitor growth of GBS in infected larvae, groups of 10 larvae were infected with 118659 (WT) and 118659ΔessC (mutant) strains (~1×10^6^ or ~1×10^8^ CFU/larvae, respectively), and bacterial burden was measured hourly in pools of larvae. As the range of infective doses for assessing the health index of the larvae was narrow (increasing the dose resulted in death of the larva, and when using smaller doses, it was hard to assess the subtle differences in the health index) we used only one infective dose for these experiments.

**Figure 6:**
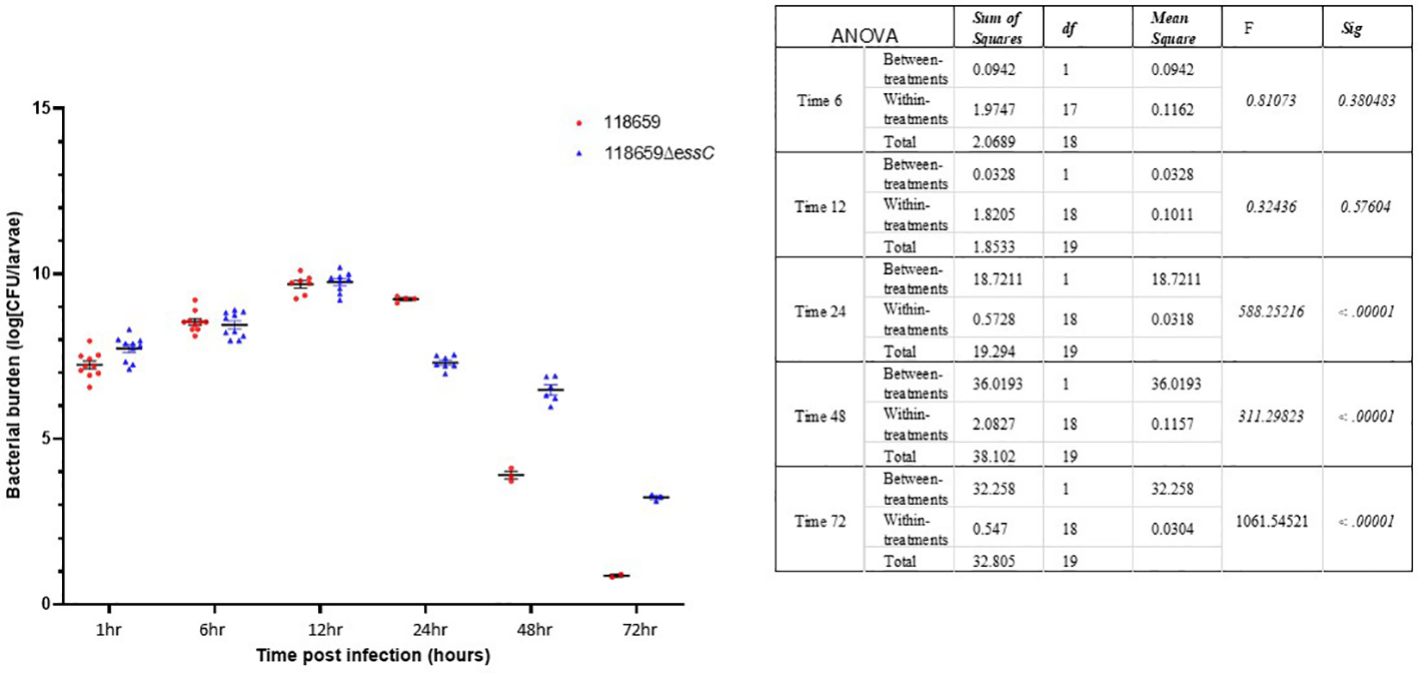
Bacterial *in vivo* counts of homogenized larvae. Bacterial counts of homogenized larvae, from two distinct experiments using three larvae per time point, showing *in vivo* growth of 118659 (WT) and mutant strain (118659Δ*essC*) in *G. mellonella* larvae following infection with sublethal dose (10^8^ or 10^6^ CFUs, respectively). Errors bars represent the standard deviation (SD). Statistics reflect the One-Way ANOVA, p<0.05.

During the first 12 hours, the larval burden of both WT and mutant isolates increased over time and reached to ~1 × 10^10^ CFU (**Figure 6**). After 12 hours, the burden of the WT strain decreased faster compared to the mutant strain. Larvae that outlived the infection with the mutant strain over 24 hours seemed to clear the GBS [10^7^ CFU/mL 24 h p.i.; 10^6^ CFU/mL 48 h p.i.]. This is probably due to efficient phagocytosis of larval hemocytes (Cutuli et al., 2019; Spencer et al., 2023). Finally, after 72 hours, the larval burden in the mutant strain was ≈ 3 logs higher compared to the WT strain (10^3^ CFU/mL to 10^1^ CFU/mL).

### *G. mellonella* health index following infection with 118659Δ*essC* strain

To measure more subtle differences in larvae health status post-infection with 118659 (WT) and 118659Δ*essC* (mutant) strains, *G*. *mellonella* larvae were monitored daily for the following attributes: activity, extent of silk production (cocoon formation) and melanization (**Table S5**). Higher activity and increased cocoon formation corresponded to healthier larva. In our experiments, the activity of the *G. mellonella* larvae was similar for both strains WT and mutant isolates. Melanin production occurred as fast as 6 hours after infection with the mutant strain and proceeded until the end of the experiments (72 hours) (**Table S5**). Melanin production was not fully correlated with mortality of the larvae. We found live larvae with full melanization even after 72 hours. Larvae infected with mutant GBS strain were able to produce more cocoon compared to larvae infected with the WT strain, even when the melanization process already started. Healthy larvae received a score of 7-8 points, while very sick larvae received a low score (<5). WT strains caused increased melanization, lower activity and cocoon formation, and were associated with a low health index – score 0 (72 h after inoculation) of *G. mellonella*. In contrast, the mutant strain caused an intermediate infectious process (72 hours after inoculation) with a health score of approximately 2.

Thus, larvae infected with mutant GBS strains received higher health scores. These larvae successfully produced cocoons, even during progressive melanization and overcame the infection.

### Decreased fitness of mutant in the *G. mellonella* model

To determine relative differences in strain fitness of 118659Δ*essC* (mutant) compared to 118659 (WT) GBS strains, we performed a competition assay using *G. mellonella*, which could be more sensitive in detecting changes in bacterial fitness, than the survival assay (Six et al., 2019). To distinguish between WT and mutant GBS strain, we induced resistance to streptomycin (Sm) in the WT strain. The mutant strain showed decreased fitness in *G. mellonella* model after 72 hours with a geometric mean of 0.4942x10^9^ CFU (geometric SD 1.1744x10^9^) versus a geometric mean of 0.8816x10^9^ CFU (geometric SD 1.1367x10^9^) in the wild type strain, p < 0.0001. The growth in the competition assay of the mutant strain was 56.2% of the growth of the wild type, a ratio of 1:1.7.As a control, to make sure that homogenization did not impact relative bacterial survival, we plated a portion of the initial mixed culture prior to injection into the larva and saw no difference in relative survival between the wild-type and the mutant strains. According to our results, there are relative differences in strain fitness of WT and mutant strain, which could confirm the decreasing virulence of the mutant strain.

### Bacterial clearance by *G. mellonella*

**Figure 7:**
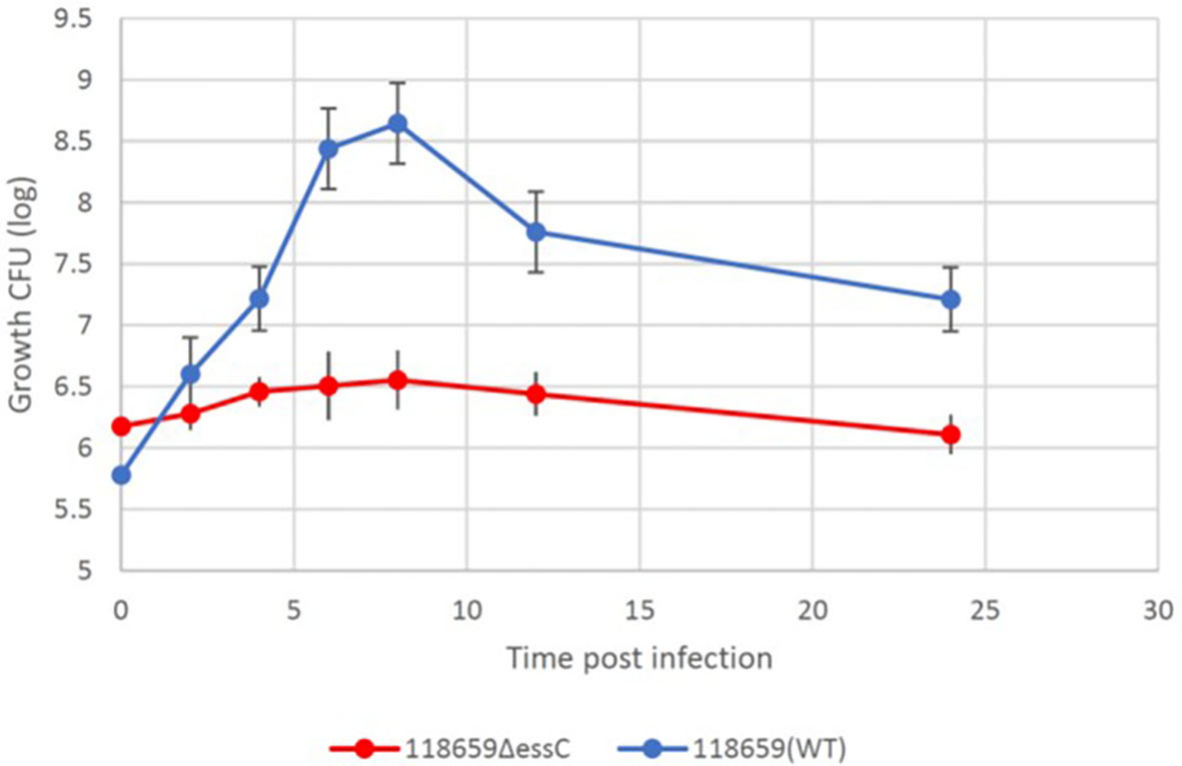
Kinetics of 118659 (WT) and 118659Δ*essC* bacterial growth *in vivo.* Larvae were injected with a sublethal inoculum and monitored for 12 hours and after 24 hours. At each fixed time point, three alive larvae were collected, kept for 15 min on ice and bathed in 70% ethanol and sterile water. The selected larvae were homogenized in two ml PBS, diluted, and plated for bacterial count in BHI with streptomycin or kanamycin. Each symbol represents a mean bacterial count obtained from tree wax worm for each time point. Statistics reflect the One-Way ANOVA, p<0.05.

To study the difference in bacterial clearance by *G. mellonella* after infection with sublethal doses of the mutant and WT strains. Larvae were injected with a sublethal inoculum ≈1 × 10^5^ CFU of 118659 (WT) strain and ≈1 × 10^6^ CFU 118659Δ*essC* of the mutant strain. The larvae were monitored every hour for 7 hours and after 12 hours. During the first 8 hours post infection, the bacterial burden of the WT in the larvae rapidly increased to three logs compared to the initial inoculum (**Figure 7**), but then the bacterial burden was reduced by 1.5 logs. In contrast, the bacterial burden of the mutant strain in the larvae failed to multiply at the same rate and eventually, the bacterial burden increased by only one log. Overall, the bacterial burden of the mutant strain was relatively stable over time. We suggest that the rapid amplification of the WT strain in the first hours post-infection may have been caused by a strong immune response, leading to decreased bacterial burden. This may explain the differences between the fitness of the WT compared to the mutant strain but warrants further study.

## Discussion

In this study we performed a genomic survey of T7SS in clinical GBS isolates, obtained from blood cultures of neonates with EOD and collected from vaginal screening of asymptomatic pregnant women. We compared the structure of T7SS locus among EOD/ST17 and colonizing/ST1 GBS isolates and identified significant differences in the structure of T7SS between EOD and colonizing GBS strains. Notably in 76.5% % of EOD/ST17 strains putative effectors were absent: *esxA* (WXG100 protein-encoding gene) and *esxB* (LXG-domain containing protein), while in colonizing/ST1 isolates three copies of the WXG100 protein-encoding gene (*esxA)* and one copy of the *essC* gene (SAG1033) were observed. In contrast to the type-specific capsular polysaccharides which are well-defined virulence determinants (Nizet and Rubens, 2014), the role of WXG100 proteins and LXG-domain containing protein as a virulence factor is not yet clearly understood. These proteins may enhance the human immune response to GBS infection. Absence of *esxA* and *esxB* genes in most ST17 isolates, may protect them from opsonization and killing by humoral and cell-mediated processes in the host. In several colonizing isolates (ST6 and ST19) structural and regulatory genes encoded by T7SS locus were missing.

Recently, two studies were published (Spencer et al., 2021; Spencer et al., 2023) in which T7SS in GBS strains was characterized. Spencer et al. (Spencer et al., 2021), suggest that T7SS comprises four subtypes based on variations in the C-terminus of *EssC* and the repertoire of downstream effectors. According to our results, subtype I is correlated to Spencer’s subtype IV, while subtype II is correlated to subtype II proposed by Spencer with slight modifications. Notably, in the subtype I that we propose, 24.5% of EOD/ST17 strains encode the WXG100 protein *esxA* (SAG1039) and have two copies of the *essC* gene. In the subtype II that we propose, the gene encoding the LXG-domain containing protein was absent in four strains. Additionally, we observed a new subtype which we named subtype III (not mentioned by Spencer’s group) in which colonizing (ST6 and ST19) isolates lack of most of the structural and regulatory genes encoded by the T7SS locus. In another study by Spencer et al. (Spencer et al., 2023) the extensive intra-subtype GBS T7SS heterogeneity was identified. In contrast to our results the homology of T7SS gene within the subtypes was very high.

In our study we highlight the diversity of T7SS in relation to clinical syndromes. We are the first to demonstrate the importance of T7SS system in EOD infections as opposed to isolates that cause colonization of pregnant women. We compared the virulence of EOD and colonizing isolates using *G. mellonella* larvae, an *in vivo* model of GBS infection. The use of *G. mellonella* larvae as bacterial infection model was developed as an alternative to murine or other vertebrate infection models to contribute to the 3Rs (reduction, replacement, and refinement) of animal use in scientific research (Cutuli et al., 2019). *In vivo* larval experiments demonstrated a difference in the pathogenicity of various clinical GBS strains. GBS strains associated with EOD demonstrated enhanced virulence in *G. mellonella* compared to colonizing strains. These results are consistent with previous study, where GBS disease associated isolates were able to establish systemic infection of *G. mellonella* larvae with extensive bacterial replication and dose-dependent larval survival (Six et al., 2019).

We further demonstrated the role of T7SS in virulence of ST17 strains and showed that it depends on the proper activity of EssC, a membrane-embedded ATPase of the FtsK/SpoIIIE family. We generated an EOD/ST17 mutans by knocking out the *essC* gene and compared the virulence of the mutant and WT strains in *G. melonella* larvae *in vivo* model. The knocked-out mutant 118659*ΔessC* had reduced ability to kill *G. mellonella*. Furthermore, LD_50_ values obtained with the 118659*ΔessC* strain were significantly higher than those obtained with the WT 118659 strain. Our results are in line with a recently published study (Spencer et al., 2021), which demonstrated that deletion of the ATPase-encoding gene, *essC*, mitigates virulence and GBS-induced inflammation in the brain, as well as cell death in brain endothelial cells in murine model of hematogenous meningitis.

Consistent with this, our data indicates that *essC* deletion affected bacterial growth during infection, as well as bacterial fitness and the response of larvae to GBS infection. We show that larvae were more effective in eradicating 118659*ΔessC* strain infection and this is probably related to different immune responses (Trevijano-Contador and Zaragoza, 2019). The competition model for *G. mellonella* is more sensitive in discerning relative differences in *Bacillus anthracis* strain fitness than the survival assay (Warne et al., 2016). In our competition assay, the mutant strain showed decreased fitness, which supports the decreased virulence of the mutant strain. The possible explanation is that T7SS in GBS secrete various effectors which induce immune tolerance against GBS infection, but further study is needed to demonstrate this assumption. In the mutant strain (118659Δ*essC*) lacking the functional secretion system, the larvae’s immune system may be more effective in eradication of the mutant strain (Trevijano-Contador and Zaragoza, 2019). Finally, we show that *essC* deletion was associated with an increase in the health index of *G. mellonella* during infection, regarding activity, cocoon formation and melaninization. The health index scoring system evaluates the health status of the larva during an infectious process. This parameter is also used to measure differences in virulence of other bacterial pathogens in *G. mellonella* (Berríos et al., 2018; Vertyporokh and Wojda, 2020). We show that melanin production by the larvae infected with the mutant strain occurred very quickly (after 6 hours p.i.). Although melaninization is usually associated with imminent death of the larva, larva remained viable, and even succeeded to produce a cocoon. We think this indicates that the mutant strain cannot succeed in causing massive dissemination of infection. This indicates that the mutant strain is attenuated compared to the WT as other parameters such as the larva’s immune function and the infective dose were similar between experiments.

In conclusion, our findings indicate that the T7SS plays a role during infection and contributes to GBS pathogenicity in isolates obtained from neonates with EOD. Knocking out the *essC* gene, considered the driver of T7SS, decreased the virulence of ST17 responsible for EOD, causing them to be less virulent compared to the virulence observed in colonizing isolates.

The proper function of T7SS, by efficient secretion of various effectors could be considered as a virulence factor of invasive GBS isolates. In most of our ST17 isolates the genes encoding to classical T7SS effector (*esxA, esxB*) were absent and further study is needed to understand the significance of this finding. Our results establish a link between T7SS and EOD in the newborn and may partially explain, why in most colonized women colonization does not proceed to infection in the newborn. Further studies are warranted to identify other effectors, their effect on substrate recognition and specificity, the inflammasome and immune response.

## Data availability statement

The sequences are available to the public at the NCBI (BioProject number ID PRJNA861829, at the following link: http://www.ncbi.nlm.nih.gov/bioproject/861829. All other data is available upon request from the corresponding author.

## Ethics statement

The studies involving human participants were reviewed and approved by Mayanei Hayeshua Medical Center Internal Review Board (approval number0023-18-MHMC). Written informed consent from the participants’ legal guardian/next of kin was not required to participate in this study in accordance with the national legislation and the institutional requirements.

## Author contributions

YS designed the experiments performed all experiments, performed the data analysis described, and wrote the article. GR contributed to the study design, experiment design, and edited the manuscript. IN contributed to data analyses, data management, in particular the data analyses of sequencing, and edited the manuscript. GV contributed to data management and analyses. MR contributed to knockout experiments. EH contributed to the knockout experiments and data analyses. DM contributed to all experiments utilizing larvae. DT-M contributed to study design. YM contributed to the idea, study design, data analyses, writing of the manuscript, and editing the manuscript. All authors contributed to the article and approved the submitted version.
