# Supplementary material for: Type VII secretion system and its effect on group B Streptococcus virulence in isolates obtained from newborns with early onset disease and colonized pregnant women
Source: Front Cell Infect Microbiol. 2023 Jul 21;13:1168530. doi: 10.3389/fcimb.2023.1168530 (PMC10400891; doi:10.3389/fcimb.2023.1168530)
Supplement: Supplementary file 1 [file DataSheet_1.docx]

**SUPPLEMENTAL MATERIAL**

**Table S1a. List of bacterial isolates used in the study**

| **Accession number** | **Specimen type** | **Serotype** | **Sequence type** | **Clonal complex** | **Expression levels** | | ***In vivo* model** |
| --- | --- | --- | --- | --- | --- | --- | --- |
|  |  |  |  |  | ***essC*** | ***esxA*** |  |
| 101298 | EOD | 3 | ST17 | CC17 | done | done |  |
| 106704 | EOD | 3 | ST17 | CC17 |  |  |  |
| 111236 | EOD | 3 | ST17 | CC17 |  |  |  |
| 112109 | EOD | 3 | ST17 | CC17 |  |  |  |
| 112767 | EOD | 3 | ST17 | CC17 |  |  |  |
| 117690 | EOD | 3 | ST17 | CC17 |  |  |  |
| 118022 | EOD | 3 | ST17 | CC17 |  |  |  |
| 118659 | EOD | 3 | ST17 | CC17 | done |  | done |
| 121684 | EOD | 3 | ST17 | CC17 | done |  | done |
| 123494 | EOD | 3 | ST17 | CC17 | done |  | done |
| 127743 | EOD | 3 | ST17 | CC17 | done | done |  |
| 127946 | EOD | 3 | ST17 | CC17 |  |  | done |
| 129618 | EOD | 3 | ST17 | CC17 |  |  |  |
| 134924 | EOD | 3 | ST17 | CC17 | done | done |  |
| 135217 | EOD | 3 | ST17 | CC17 | done |  |  |
| 139904 | EOD | 3 | ST17 | CC17 |  |  |  |
| 139934 | EOD | 3 | ST17 | CC17 | done | done |  |
| M38307 | Colonization | 6 | ST1 | CC1 | done | done | done |
| M38346 | Colonization | 3 | ST19 | CC17 |  |  |  |
| M38421 | Colonization | 6 | ST1 | CC1 | done | done | done |
| M38603 | Colonization | 6 | ST19 | CC17 |  |  |  |
| M38742 | Colonization | 6 | ST1 | CC1 | done | done | done |
| M38839 | Colonization | 6 | ST6 | Singleton |  |  |  |
| M38914 | Colonization | 6 | ST1 | CC1 | done | done | done |
| M39081 | Colonization | 6 | ST1 | CC1 | done |  |  |
| M39881 | Colonization | 6 | ST1 | CC1 | done |  |  |
| M40042 | Colonization | 3 | ST1 | CC1 | done |  |  |
| M40064 | Colonization | 6 | ST1 | CC1 | done |  |  |
| M40083 | Colonization | 3 | ST19 | CC17 |  |  |  |
| M40942 | Colonization | 6 | ST1 | CC1 |  |  |  |
| M41387 | Colonization | 6 | ST1 | CC1 |  |  |  |
| M41827 | Colonization | 6 | ST1 | CC1 |  |  |  |
| W19655 | Colonization | 6 | ST1 | CC1 |  |  |  |

The table describes the list of bacterial isolates used in this study and the experiments performed on these isolates. EOD – early onset disease of the newborn.

**Table S1b. List of plasmids and bacterial strain used for the knock out procedure**

| **Reference** | **Description** | **Plasmid** |
| --- | --- | --- |
| Promega | A commercial T-vector for AT cloning | pGEM-T® Easy |
| (Perez-Casal et al., 1991) | *Streptococcus-E. coli* temperature sensitive shuttle vector | pJRS233 |
| This study | pGEM-T® Easy containing a 3809-bp bp fragment of *essC* gene | pGEM:*essC* |
| This study | pGEM-T® containing an ΩKm resistance cassette, flanked by 2934 bp fragment of *essC* gene | pGΔ*essC*ΩKm |
| This study | pJRS233 containing an ΩKm resistance cassette, flanked by 2954 bp *essC* gene | pJΔ: *essC*: ΩKm |
| **Source** | **Description** | **Strain** |
| Invitrogen | A cloning *E. coli* strain | DH5α |

**Table S2. Oligonucleotides used for PCR analysis of mutant GBS strain**

| Sequence | Primer name |
| --- | --- |
| ACAAGTGTCCGTGATTCAGAGT | EssC-KO |
| ACGACCAAGGGATTGAAG | Conf_KO_essC |
| GACGTGCTAAGTCCAATG | Conf_KO_essC |
| GCGAGCAGGGGAATTGATCC | v-omega-Km1 |
| TTACTAAGCTGATCCGGTGG | v-omega-Km2 |

**Table S3: Health index scoring system**

| Category | Description | Score |
| --- | --- | --- |
| Activity | No activity | 0 |
|  | Minimal activity on stimulation | 1 |
|  | Active when stimulated | 2 |
|  | Active without stimulation | 3 |
| Cocoon formation | No cocoon | 0 |
|  | Partial cocoon | 0.5 |
|  | Full cocoon | 1 |
| Melanization | Complete melanization (black) | 0 |
|  | Dark spots on brown larvae | 0 |
|  | ≥3 spots on beige larvae | 2 |
|  | <3 spots on beige larvae | 3 |
|  | No melanization | 4 |

**Table S4: List of primer used for RT-qPCR of T7SS genes**

| Sequence (5’-3’) | Direction | Primer name | Gene name |
| --- | --- | --- | --- |
| CATGCATATGGATCACGCGA | F | SAG1039 | *esxA* |
| CCTGCAAAAGATGGCGCAAT | R |  |  |
| ATGGTTCGTACTTTGGTCGTGCTT | F | SAG1033 | *essC* |
| TCGGCCTGTTTCAACTTTTCGCT | R |  |  |
| AATTAGTCCGTTCTCCTGGTGTTT | F | rpoB | *rpoB* |
| ACGTGTGCGGTCGATACGT | R |  |  |
| CTGGACGGTTAATGGTAAGC | F | esaA-F | *esaA* |
| CTTCTGCCTCCAAGTAAGAC | R | esaA-R |  |
| ATGTTGGCAGCGGATACTTC | F | essB-F | *essB* |
| GGCCAACTCGTACTTCTGTG | R | essB-R |  |
| TTCAAACTGAATTAACTAAAG | F | essA-F | *essA* |
| TTCATCCTTTTTGTCCTTTAT | R | essA-R |  |

**Table S5: *G. mellonella* health status index following infection with WT and the mutant isolates**

|  | Activity | | Cocoon formation | | Melanization | |
| --- | --- | --- | --- | --- | --- | --- |
| Hours | **118659∆*essC*** | **118659** | **118659∆*essC*** | **118659** | **118659∆*essC*** | **118659** |
| 0 | 3 | 3 | 2 | 2 | 4 | 4 |
| 6 | 3 | 3 | 2 | 1 | 3 | 4 |
| 12 | 2 | 2 | 2 | 0 | 2 | 4 |
| 24 | 2 | 2 | 2 | 0 | 0 | 3 |
| 48 | 1 | 1 | 1 | 0 | 0 | 1 |
| 72 | 1 | 0 | 1 | 0 | 0 | 0 |

Evaluation of *G. mellonella* health status post infection with 10^7^ CFU of the 118659 WT and the mutant (118659*∆EssC*) strains. *G. mellonella* was monitored for 72 hours. At each time (at 6, 24 h, 48 h and 72 h post infection) a score was provided for each attribute (activity, cocoon formation and melanization) of an individual larva. Each time point represents the combined (additive) score data of 10 larvae from three independent experiments and are presented as mean.

**Legends to Supplement figures**

**Legend to figure S1:** Schematic drawing showing the replacement of the chromosomal *essC* gene with the kanamycin resistance cassette (*KmR*), the location of the primers EssC-KO-F and EssC-KO-R used for PCR amplification of the regions spanning the deleted fragment; the primers for kanamycin resistance v-omegaKm1 and v-omegaKm2, and a pair of primers from inner part of *essC* gene (Conf-KO-essC), which should have been replaced by the omega kanamycin cassette

**Legend to figure S2**: . **(a)** Kaplan–Meier survival curves of *G. mellonella* challenged with serial dilutions of four colonizing isolates. **(b)** Four EOD isolates. All graphs show the combined survival curves of three independent experiments, with use of 10 larvae/group. PBS-injected larvae were used as a negative control, and demonstrate a dose-dependent survival curves.

**Legend to figure S3:** EOD isolates showed increased virulence in *G. mellonella*. LD_50_ values were determined by Probit analysis following infection of larvae by four EOD isolates (blue) and four colonizing isolates (red). Each data point represents the LD_50_ of each experiment in which groups of 10 larvae were infected with four different inoculums, analyzed with the Mann-Whitney test (p<0.0001).

**Legend to figure S4.**  Knock out confirmation of *essC* gene by PCR method.

**(a)** Knock out was confirmed by using EssC-KO -F and EssC-KO -R primers flanking the *essC* gene. Lane 3 -118659 WT strain (~ 3300 bp.); Lane 4 - 118659∆essC (2900 bp.); Lane 6 – 1 kb DNA Ladder. (b) Lane (2,4): 118659∆essC mutant, with primers EssC-KO- F flanking the *essC* gene and v-omegaKm1 flanking the kanamycin resistance cassette (~ 621 bp.); Lane (6,8): 118659∆essC strain, with primers EssC-KO- R flanking the *essC* gene and v-omegaKm2 flanking the kanamycin resistance cassette (~ 574 bp). For comparison, the absence of band was identified with 118659 WT strain (Lane 3 and 7).

**Legend to figure S5**. Growth rate of the wild-type 118659 (WT) (blue line) and 118659∆*essC* (red line) strains in BHI medium for 8 hours. Assays were repeated three independent times. Statistics reflect the one-way ANOVA and are presented as mean ± SD, p<0.001.

**Legend to figure S6**. Cultures of 118659∆*essC* and 118659 (WT) strains were grown overnight on blood agar plate. Similar hemolysis of GBS colonies was observed for WT and mutant strain.

**Legend to figure S7:** LD50 of the 118659(WT) and the mutant 118659∆*essC* isolates in *G. mellonella* model. LD_50_ values determined by Probit analysis 118659∆*essC* (red) and 18659WT (blue), and analyzed with the Mann-Whitney test, p<0.001.

**Figure S1:** The schematic representation of pJRS EssC omega Km plasmid which was transformed to *E. coli* DH5α


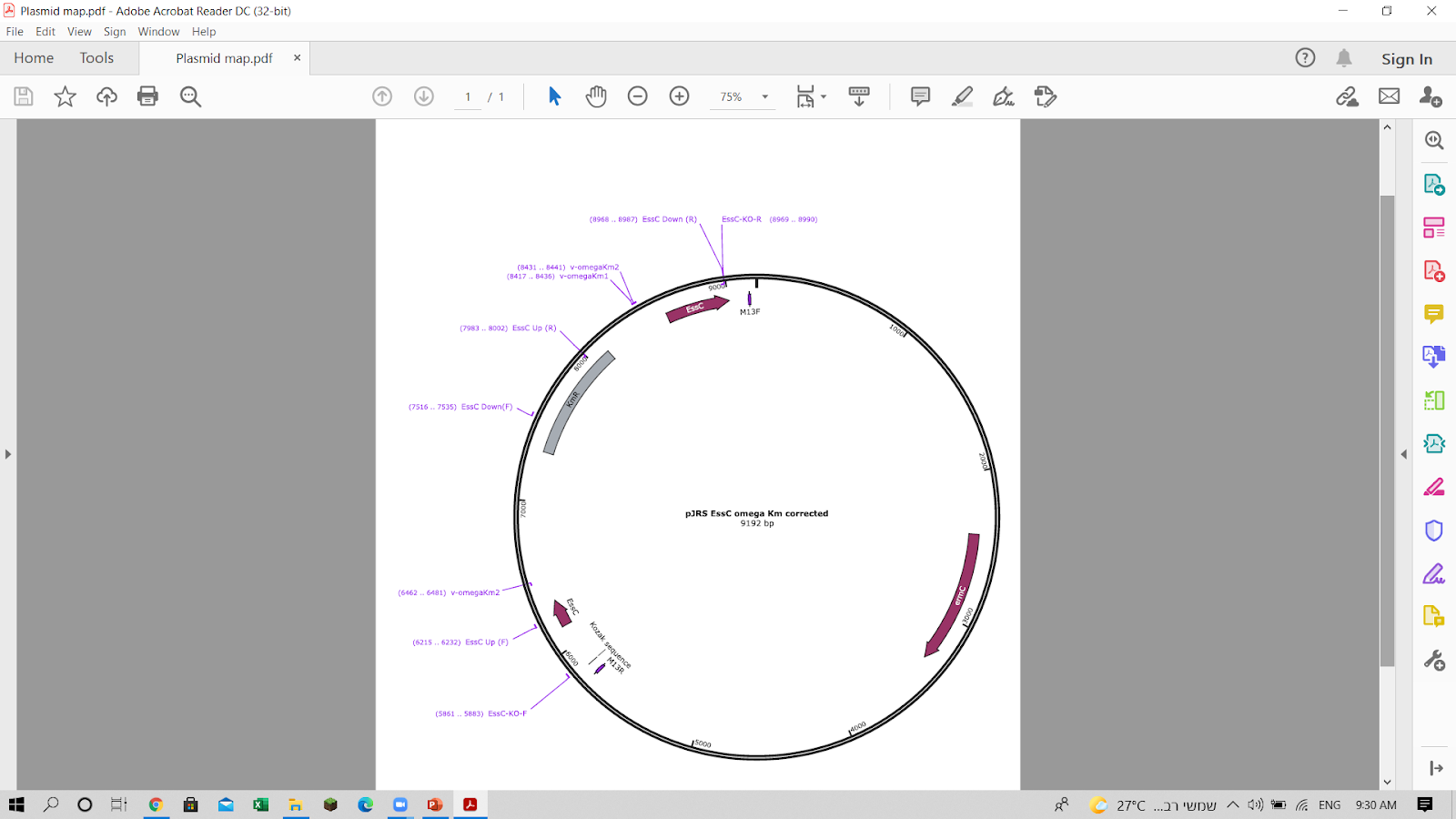


**Figure S2:** *G. mellonella* challenged with serial dilutions of *S. agalactiae* EOD strain (a) and colonizing strain (b), demonstrate a dose-dependent survival

**a.**
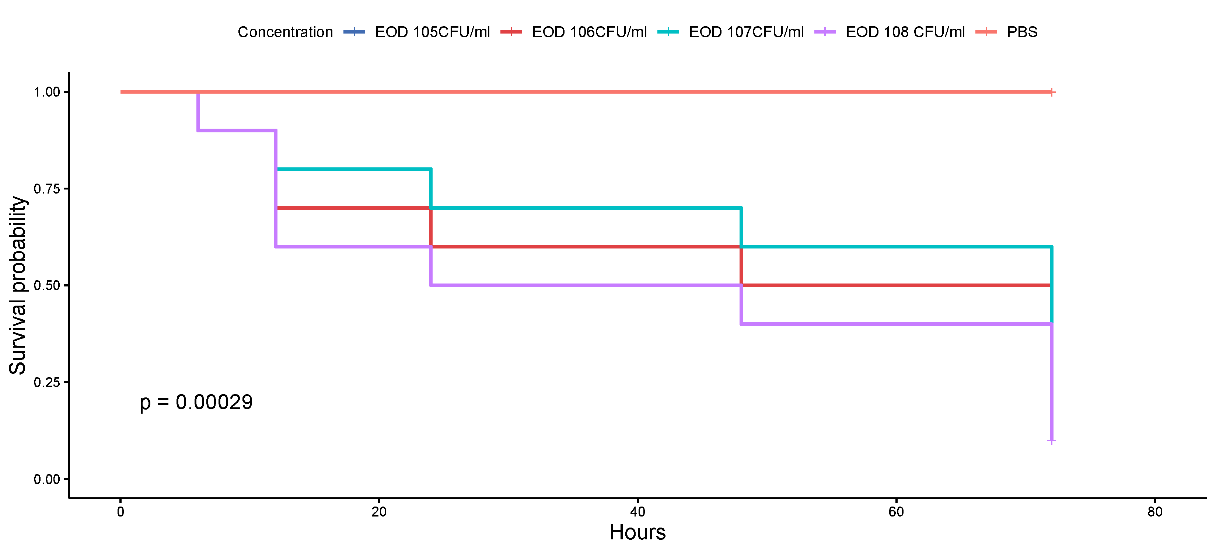


**b.**
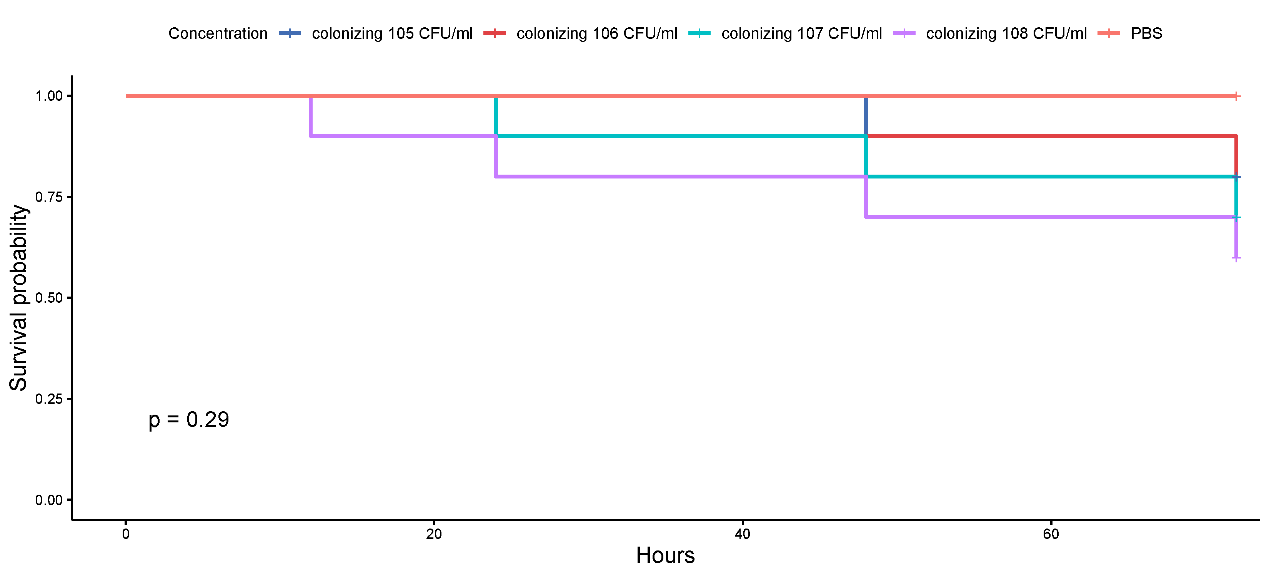


**Figure S3:** LD_50_ values of EOD isolates and colonizing isolates in *G. mellonella* model.

**
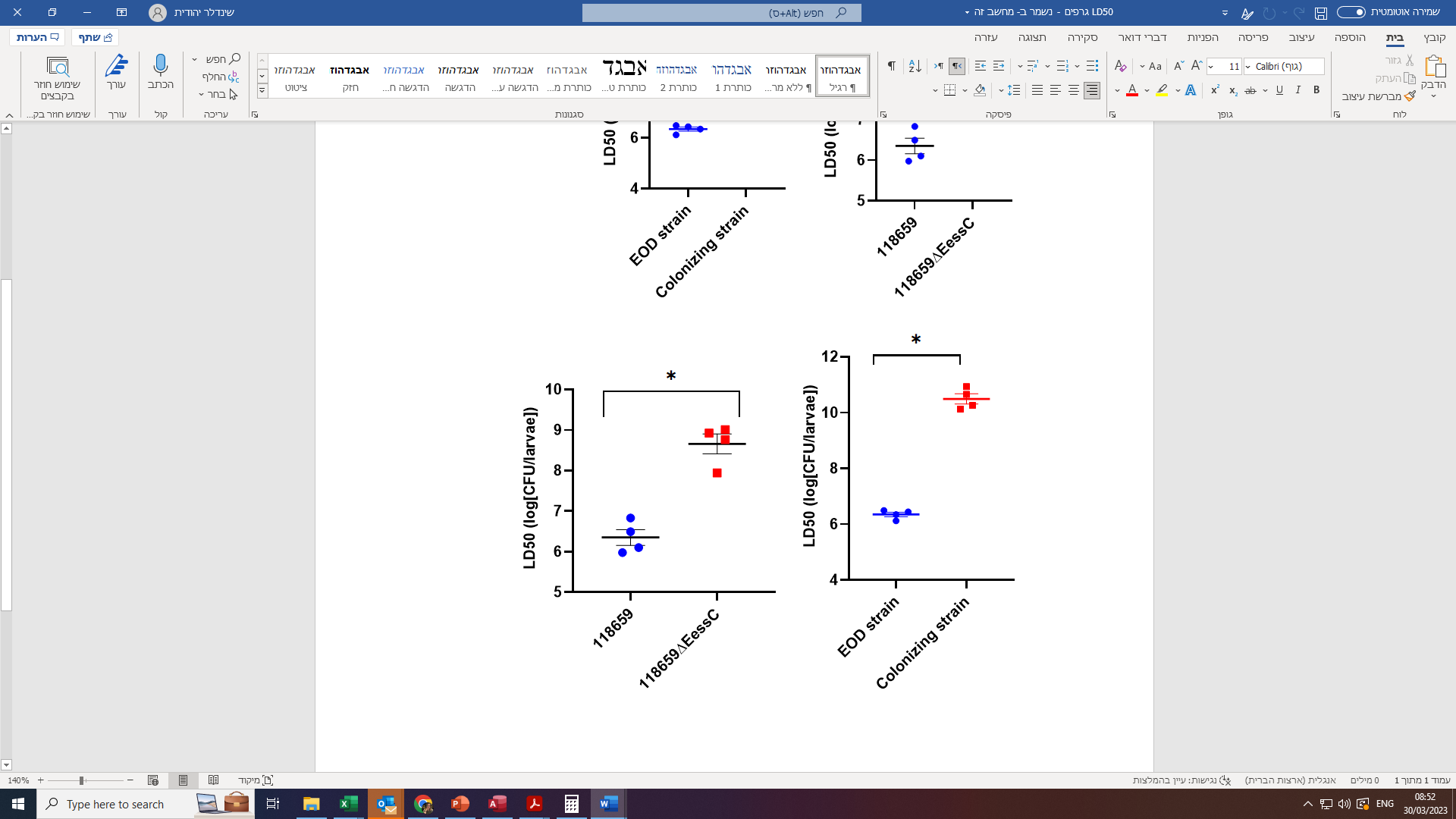
**

**Figure S4:** PCR analysis of 118659Δ*essC* mutant knockout


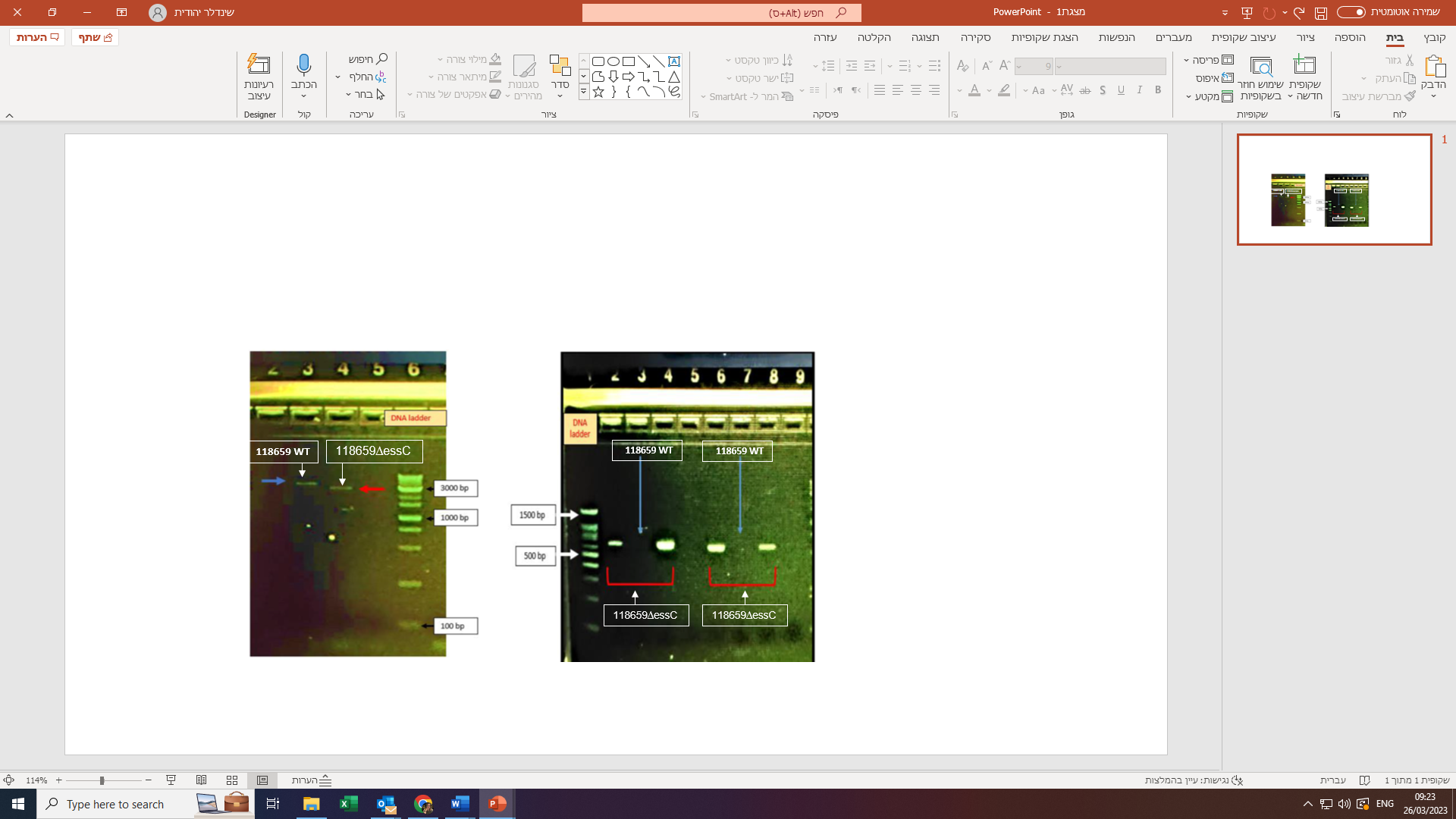


**Figure S5:** Growth rate of the wild-type 118659 (WT) and the mutant 118659Δ*essC* isolates


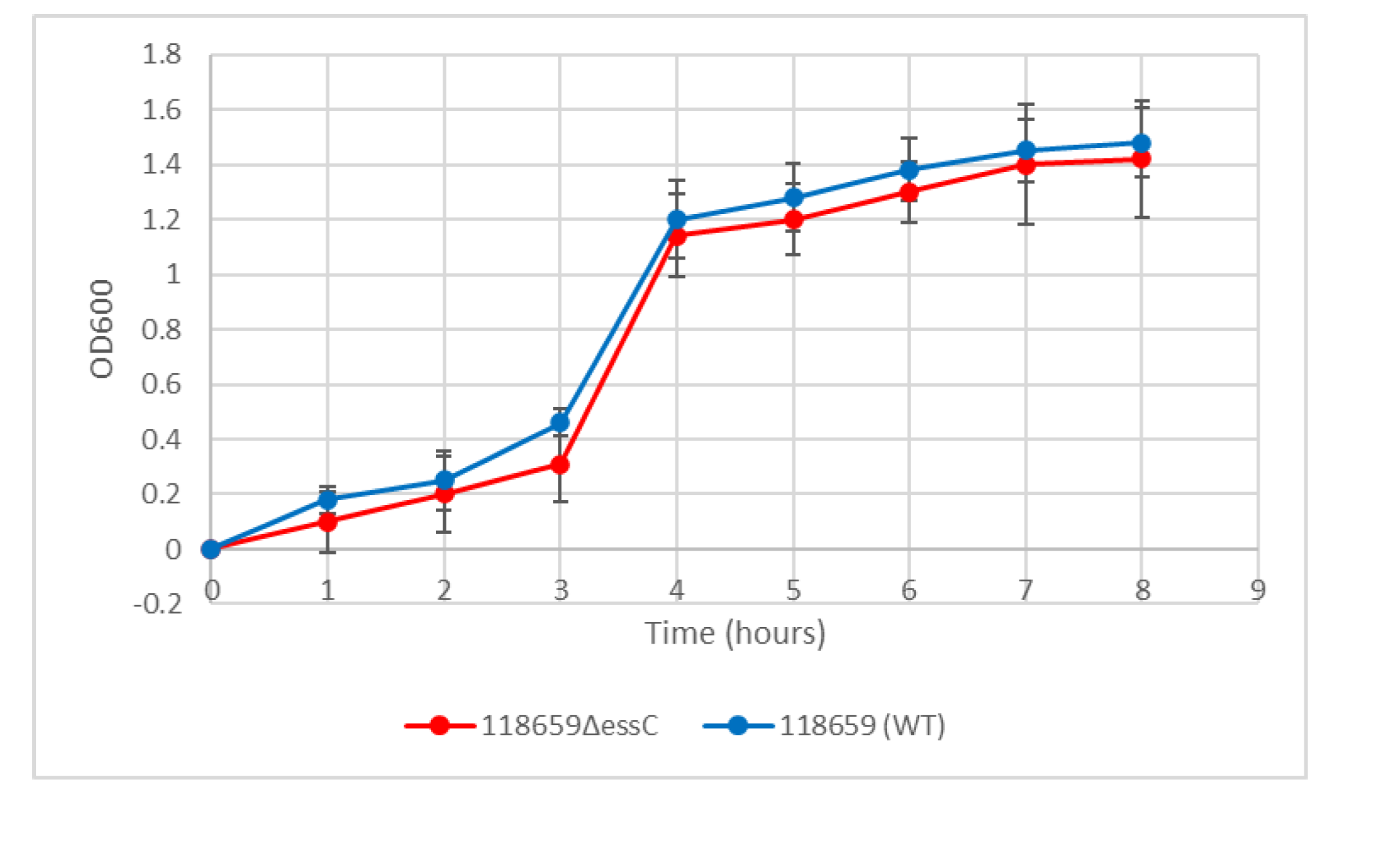


**Figure S6:** Hemolysis pattern of 118659∆*essC* (on the left side) and 118659 (WT) strains (on the right side) plated on blood agar after overnight incubation.


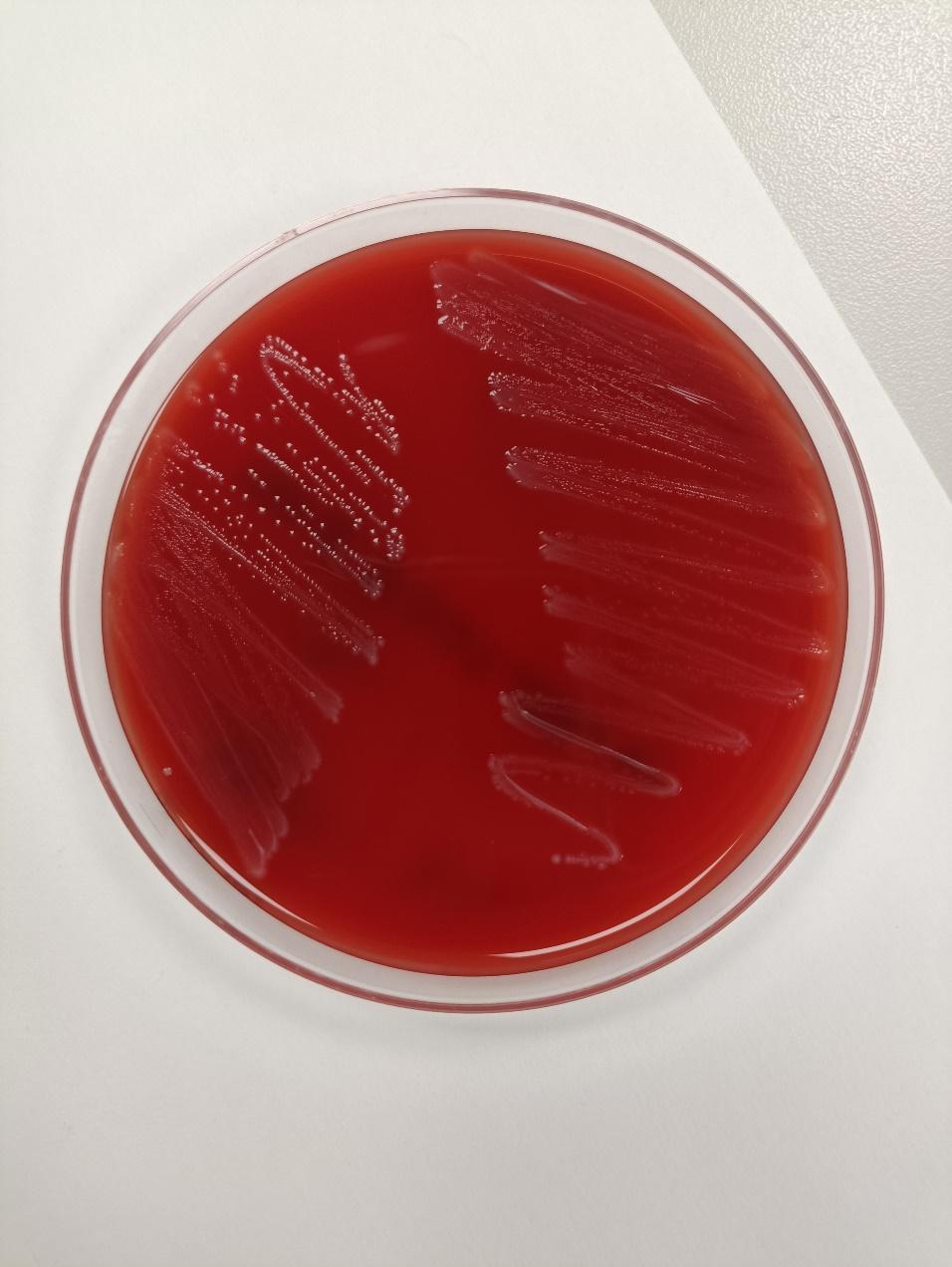


**Figure S7:** *G. mellonella* challenged with serial dilutions of (a) 118659 WT strain; (b) 18659∆*essC* colonizing strain, demonstrate a dose-dependent survival; (c) LD_50_ of the 118659(WT) and the mutant 118659∆*essC* isolates in *G. mellonella* model

**(a)**
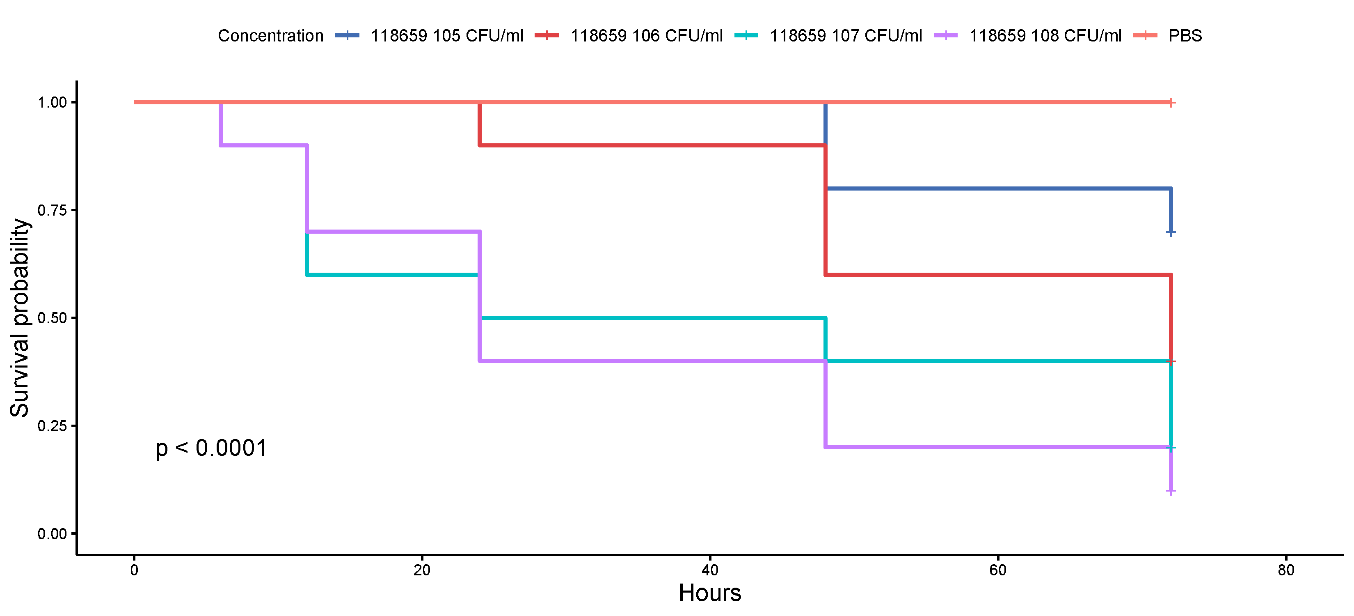


**(b)**
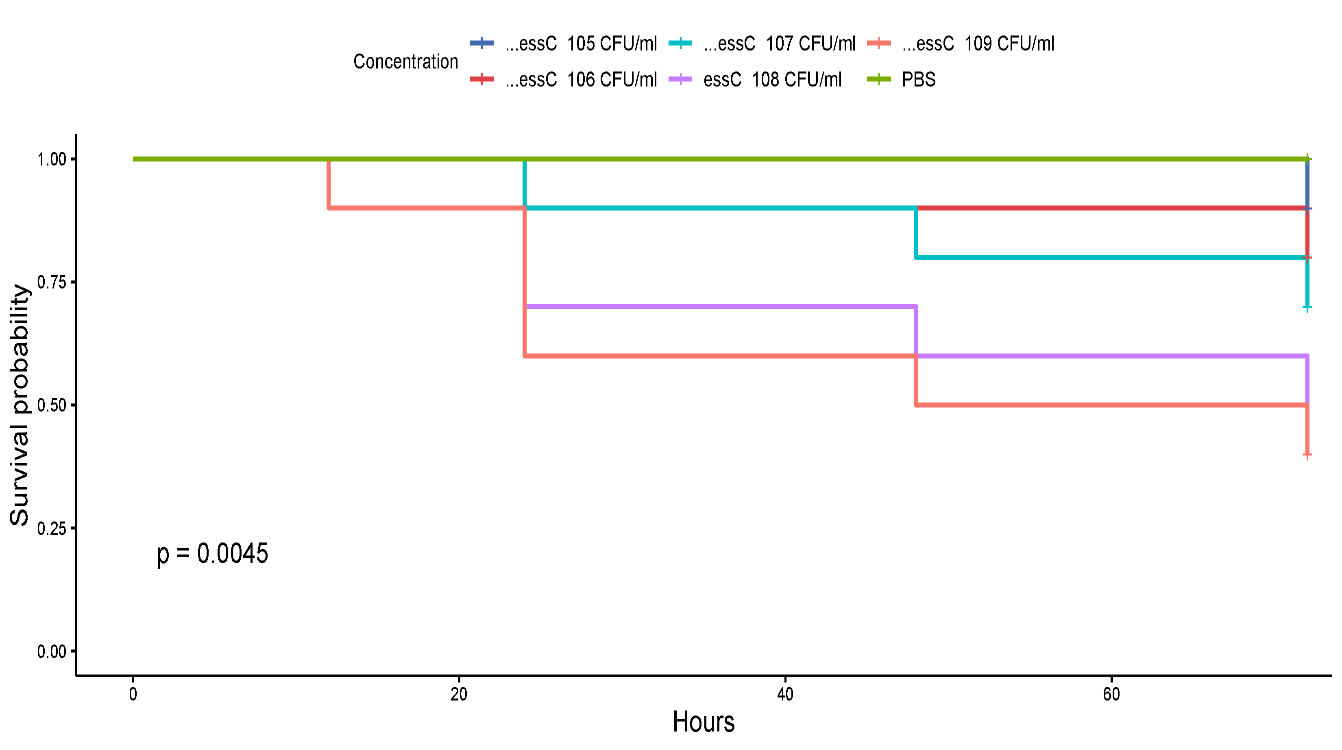


**
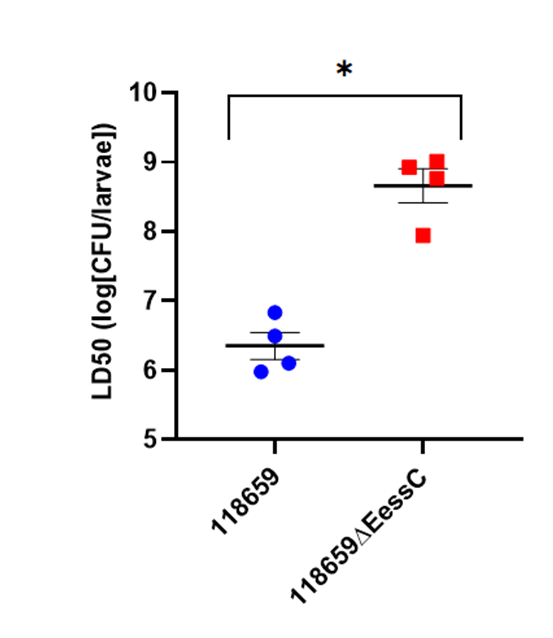
(c)**
